# Bi-component T1ρ and T2 Relaxation Mapping of Skeletal Muscle *In-Vivo*

**DOI:** 10.1038/s41598-017-14581-9

**Published:** 2017-10-26

**Authors:** Azadeh Sharafi, Gregory Chang, Ravinder R. Regatte

**Affiliations:** Bernard and Irene Schwartz Center for Biomedical Imaging, Department of Radiology, New York University School of Medicine, New York, NY USA

## Abstract

The goal of this paper was to evaluate the possibility of bi-component T1ρ and T2 relaxation mapping of human skeletal muscle at 3 T in clinically feasible scan times. T1ρ- and T2-weighted images of calf muscle were acquired using a modified 3D-SPGR sequence on a standard 3 T clinical MRI scanner. The mono- and biexponential models were fitted pixel-wise to the series of T1ρ and T2 weighted images. The biexponential decay of T1ρ and T2 relaxations was detected in ~30% and ~40% of the pixels across all volunteers, respectively. Monoexponential and bi-exponential short and long T1ρ relaxation times were estimated to be 26.9 ms, 4.6 ms (fraction 22%) and 33.2 ms (fraction: 78%), respectively. Similarly, the mono- and bi-exponential short and long T2 relaxation times were 24.7 ms, 4.2 ms (fraction 15%) and 30.4 ms (fraction 85%) respectively. The experiments had good repeatability with RMSCV < 15% and ICC > 60%. This approach could potentially be used in exercise intervention studies or in studies of inflammatory myopathies or muscle fibrosis, permitting greater sensitivity and specificity via measurement of different water compartments and their fractions.

## Introduction

Skeletal muscle is a very heterogeneous tissue, which composed of different types of muscle fiber. In most cases, the muscular disease affects the properties of the muscle fibers and as a result, changes their relaxation times. For example, in muscle fibrosis, the damaged striated skeletal muscle is replaced mainly by excessive collagen^1,2^. The change of muscle fiber type affects the muscle relaxation times^3,4^. As the disease progresses, the water content of the muscle will increase, leading to a lengthening of the relaxation time^3,4^. Hence, monitoring relaxation times such as T2 and T1ρ as noninvasive biomarkers may provide valuable information on the disease progression. Monoexponential measurement of T1ρ and T2 was investigated for disease monitoring^5–9^ and the elevated T1ρ and T2 due to a disease or an injury have been shown in several studies^10–12^. Recently, Iijima *et al*.^10^ showed the increase of T2 relaxation time in rotator cuff muscles with the extent of the tear. Moreover, Maillard *et al*.^7^ proposed using T2 relaxation as a quantitative measure of muscle inflammation. In another study, Hatakenaka *et al*.^13^ showed the effect aging on T2 relaxation time. However, as shown by Hazlewood *et al*.^14^ different fractions of non- (or slowly) exchanging water exists in the muscle tissues. Hence; a multiexponential model may better present the relaxation time components in the muscle. Saab *et al*. reported the multiexponential behavior of T2 relaxation in skeletal muscle. More recently, Araujo *et al*.^2^ proposed a method to measure the short T2 component in skeletal muscle (SKM) in the presence of fat using a UTE sequence. Moreover, the biexponential behavior of T1ρ relaxation has been observed in rat muscle^15^.

To the best of our knowledge, the biexponential measurement of T1ρ and T2 has yet to be reported *in vivo*. The purpose of this work is to evaluate the *in-vivo* feasibility of biexponential analysis of T1ρ and T2 relaxation times of human calf muscle using 3T MRI in clinically feasible scan times.

## Results

### Monte Carlo Simulation

**Figure 1:**
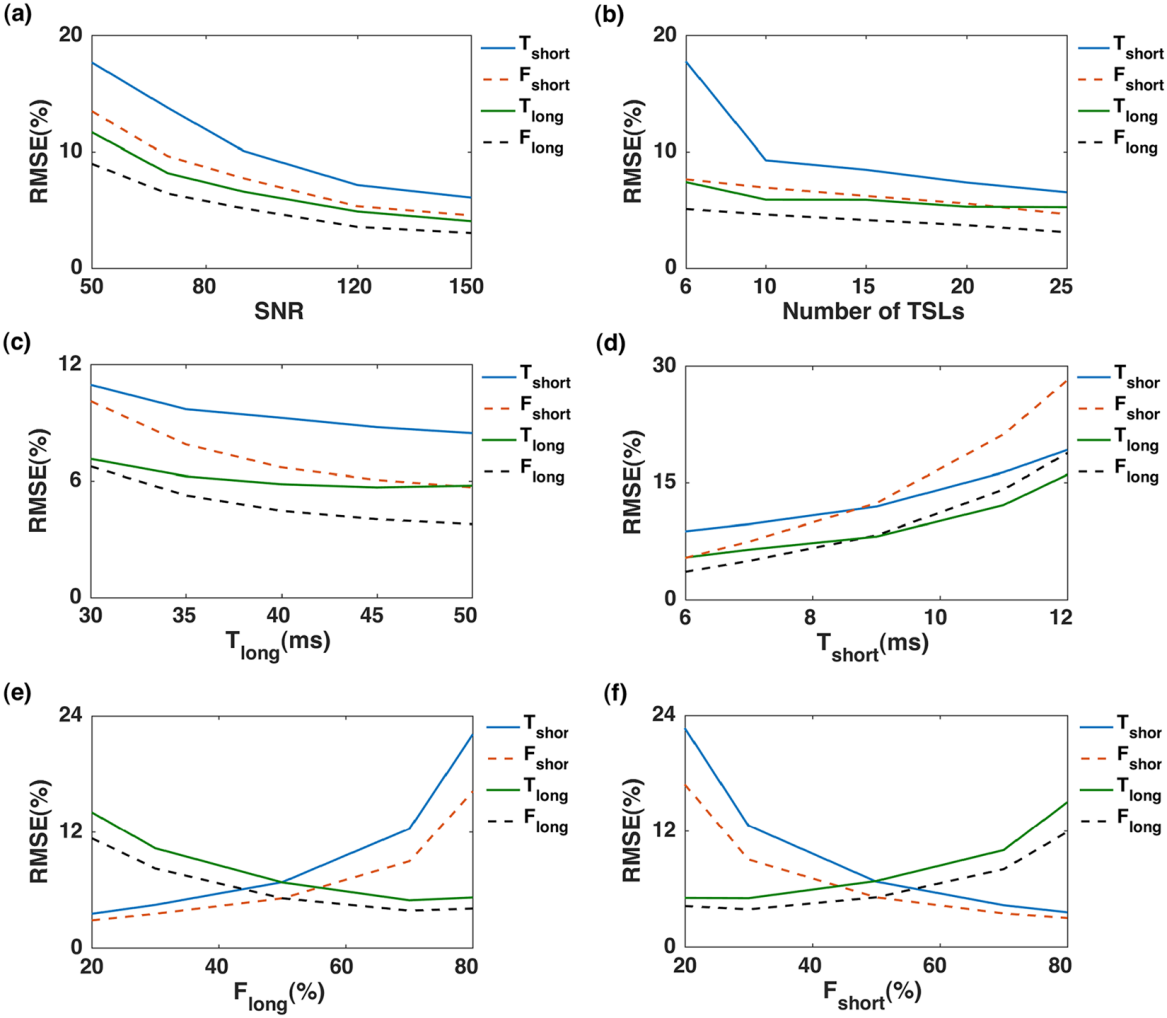
Monte Carlo Simulation. (**a**) The estimation error decrease with higher SNR (**b**) Acquiring more time points decreases the estimation error. (**c**,**d**) The estimation error is higher for shorter long (**c**) and longer short (**d**) components. (**e**,**f**) The component with higher fraction can be estimated better than the one with lower fraction.

As shown in Fig. 1, higher SNR leads to more accurate estimation (Fig. 1a). The SNR of the *in-vivo* T1ρ and T2 experiments was 65 ± 11 and 76 ± 13, respectively; hence about [12–16%] (T1ρ) and [9–14%] (T2) estimation error was expected for *in-vivo* estimation of short relaxation time, and about [5–10%] error is expected in estimating the long relaxation time as well as the short and long fractions. The number of time points (TSL or TE) also affects the estimation. As shown in Fig. 1b, acquiring data in more time points increases the fitting accuracy and as a result better relaxation estimation (Fig. 1b). However, the acquisition time increases linearly with the number of points, so a trade-off must be made between image acquisition time and measurement accuracy. As shown in Fig. 1b, the improvement from 10 to 15-time points is negligible (less than 2%) considering the 50% increase in the total scan time. Hence, in this study, 10 *TSL/TE* points were selected for the *in-vivo* experiment. As shown Fig. 1c and d, the estimation errors are higher for shorter long and longer short components. The effect of component fractions on the estimation is shown in Fig. 1e and f. The component with higher fraction is estimated more accurately than the one with a smaller fraction.

### *In vivo* experiment

**Figure 2:**
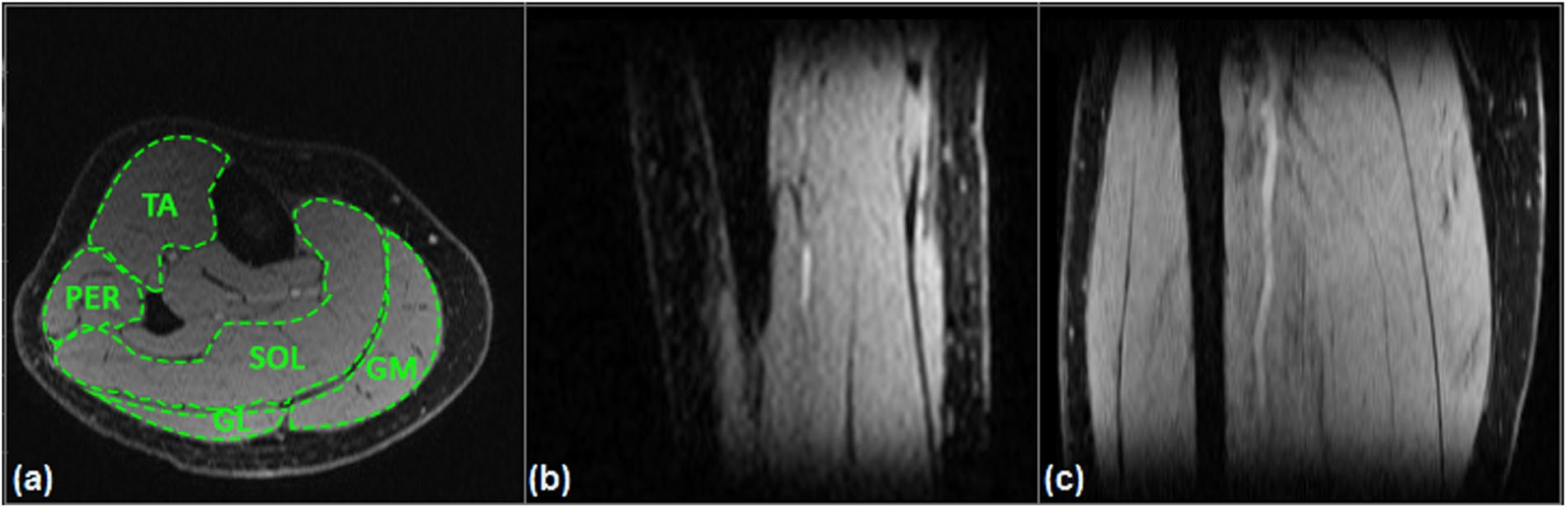
A representative T1ρ scan in (**a**) axial (**b**) sagittal and coronal plane at TSL = 02 ms. The calf muscle ROIs: Gastrocnemius Medialis (GM), Gastrocnemius Lateralis (GL), Soleus (SOL), Peroneus longus (PER), and Tibialis Anterior (TA).

**Figure 3:**
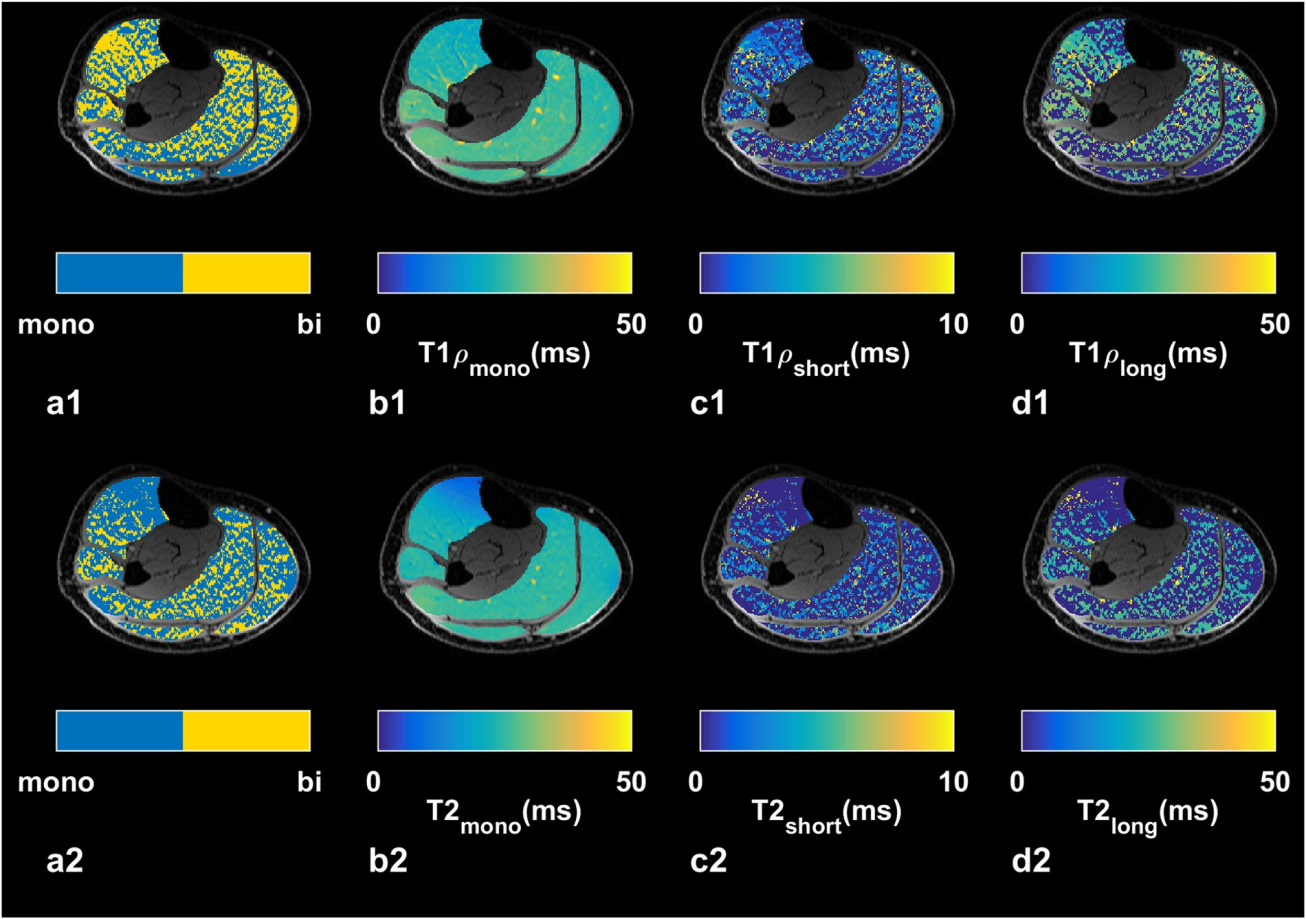
A representative example of T1ρ (a1–d4) and T2 (a2–d2) relaxation maps. (**a**) Binary maps show the location of excluded pixels in biexponential maps. (**b**) Monoexponential relaxation maps. (**c**) Biexponential short and (**d**) long relaxation maps.

**Figure 4:**
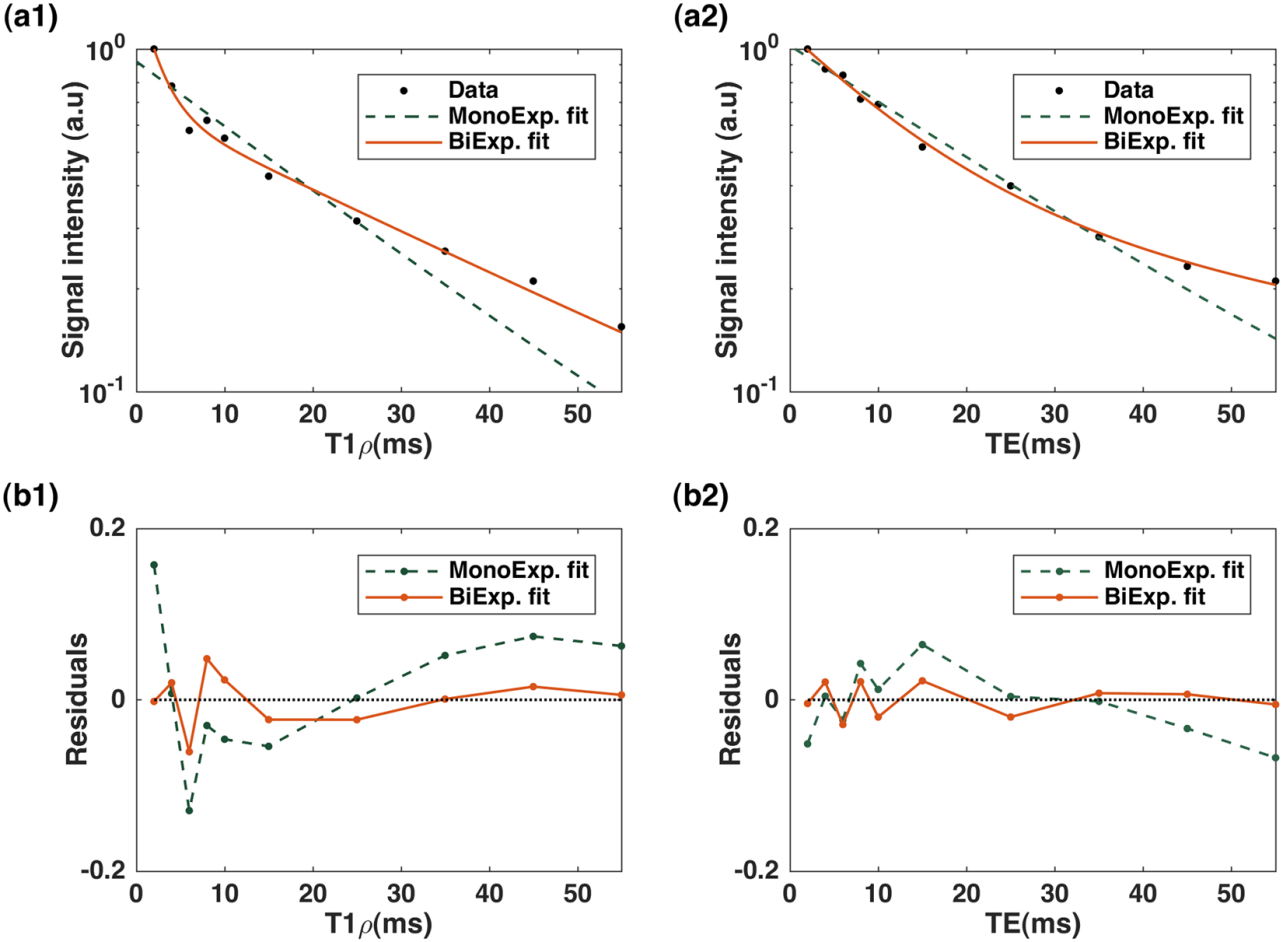
Mono- and bi-exponential fit comparison. (**a1**,**b1**) T1ρ and (**a2**,**b2**) T2 decay and the fit residuals in representative voxels.

Figure 2 shows a representative slice of a scan with TSL = 2 ms in axial, sagittal, and coronal planes and the regions of interests (ROIs) in which the relaxation components were estimated. A representative example of T1ρ and T2 maps are shown in Fig. 3a 1–d1 and Fig. 3a 2–d2 respectively. The summary of descriptive statistics calculated across eight participants in each ROI is summarized in Table 1. The estimated monoexponential and biexponential short and long T1ρ over different regions were varied between [23.7 ms, 31.4 ms], [3.5 ms–13.5 ms] and [28.1 ms–41.3 ms], respectively; while the T2 mono, short and long components ranged from [18.5 ms–27.8 ms], [4.2 ms–8.7 ms] and [28.4–55.2 ms], respectively. The short component has lower fraction than the long relaxing component. In this study, we observed that a biexponential fit is a better describes the T1ρ and T2 relaxation decay than a monoexponential model. As shown in Fig. 4a, since the monoexponential fit appears as a straight line in logarithmic scale, the deviation of the data points from the line indicates the existence of more than one exponential term^16^ in the model. Moreover, the biexponential fit has smaller residuals than the monoexponential fit which confirm that it can better represent the relaxation decay.

**Table 1:** Summary of T1ρ and T2 relaxation times estimation in 5 different regions of interest.

ROI	Relaxation Type	T_mono_ (ms)	T_short_ (ms)	F_short_ (%)	T_long_ (ms)	F_long_ (%)	Ratio (%)
GM	T1ρ	28 ± 1.5	6.1 ± 0.6	21 ± 1.5	36 ± 2.3	79 ± 1.5	30 ± 0.1
	T2	25 ± 0.9	6.3 ± 0.7	18 ± 4.5	34 ± 2.6	82 ± 4.5	18 ± 0.0
GL	T1ρ	28 ± 2.2	7.8 ± 2.9	25 ± 15	42 ± 11	75 ± 15	22 ± 0.1
	T2	26 ± 1.1	5.9 ± 0.8	15 ± 3.7	33 ± 3.5	85 ± 3.7	17 ± 0.1
SOL	T1ρ	29 ± 1.0	6.4 ± 0.6	21 ± 2.5	37 ± 2.3	79 ± 2.5	30 ± 0.1
	T2	26 ± 0.6	5.6 ± 0.3	16 ± 2.4	33 ± 1.1	84 ± 2.4	16 ± 0.0
PER	T1ρ	28 ± 2.6	6.2 ± 1.3	26 ± 10	37 ± 5.0	74 ± 10	32 ± 0.1
	T2	25 ± 2.2	5.9 ± 1	22 ± 15	35 ± 8.2	78 ± 15	20 ± 0.1
TA	T1ρ	25 ± 1.0	6.2 ± 0.4	31 ± 5.8	37 ± 1.3	69 ± 5.8	28 ± 0.1
	T2	23 ± 2.2	6.8 ± 1.0	30 ± 10	39 ± 5.3	70 ± 10	14 ± 0.1
Global	T1ρ	27 ± 1.2	6.3 ± 0.3	25 ± 2.7	37 ± 1.5	75 ± 2.7	29 ± 0.0
	T2	25 ± 1.0	6.1 ± 0.5	20 ± 3.6	35 ± 2	80 ± 3.6	17 ± 0.0

### Statistical Analysis

**Figure 5:**
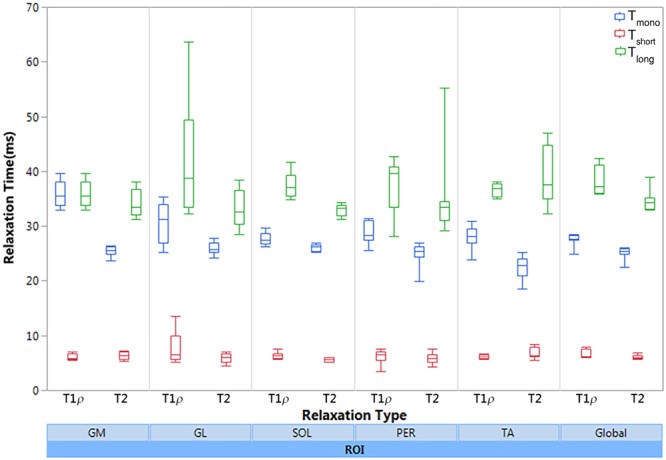
T1ρ and T2 relaxation times comparison in different muscle ROIs.

Figure 5 shows the comparison between T1ρ and T2 relaxation time in different muscle ROIs. The Wilcoxon rank sum test results showed that the global mono and long T1ρ relaxation components were significantly higher than T2 relaxation. The gender difference analysis results for T1ρ and T2 relaxation components revealed that the short T2 relaxation component was significantly greater in male participants than the female participants. In addition, the Kruskal-Wallis test was applied to investigate the difference between different ROIs. The results showed that there is a statistically significant difference in monoexponential T1ρ (P < 0.001) and T2 (P = 0.0038) in different ROIs. No significant difference was observed in biexponential components. The pairwise comparison between ROIs is shown in Table 2.

**Table 2:** Pairwise T1 and T2 comparison between different muscle ROIs. The statistically significant different ROI p-values are shown with an asterisk.

ROI	-ROI	T1ρ_mono_ p-Value	T1ρ_short_ p-Value	T1ρ_long_ p-Value	T2_mono_ p-Value	T2_short_ p-Value	T2_long_ p-Value
GM	GL	0.01*	0.2	0.2	0.2	0.4	0.1
GM	SOL	0.001*	0.3	0.3	0.4	0.6	0.8
GM	PER	0.001*	0.4	0.4	0.9	0.4	0.6
GM	TA	0.001*	0.5	0.5	0.004*	0.1	0.4
GL	SOL	0.2	0.6	0.5	0.003*	0.4	0.9
GL	PER	0.4	0.8	1	0.8	0.1	0.04*
GL	TA	0.2	0.6	0.9	0.4	0.9	0.7
SOL	PER	0.3	0.8	0.8	0.3	0.005*	0.005*
SOL	TA	0.6	0.6	0.6	0.002*	0.6	0.7
PER	TA	0.5	0.4	0.4	0.024*	0.2	0.1

**Figure 6:**
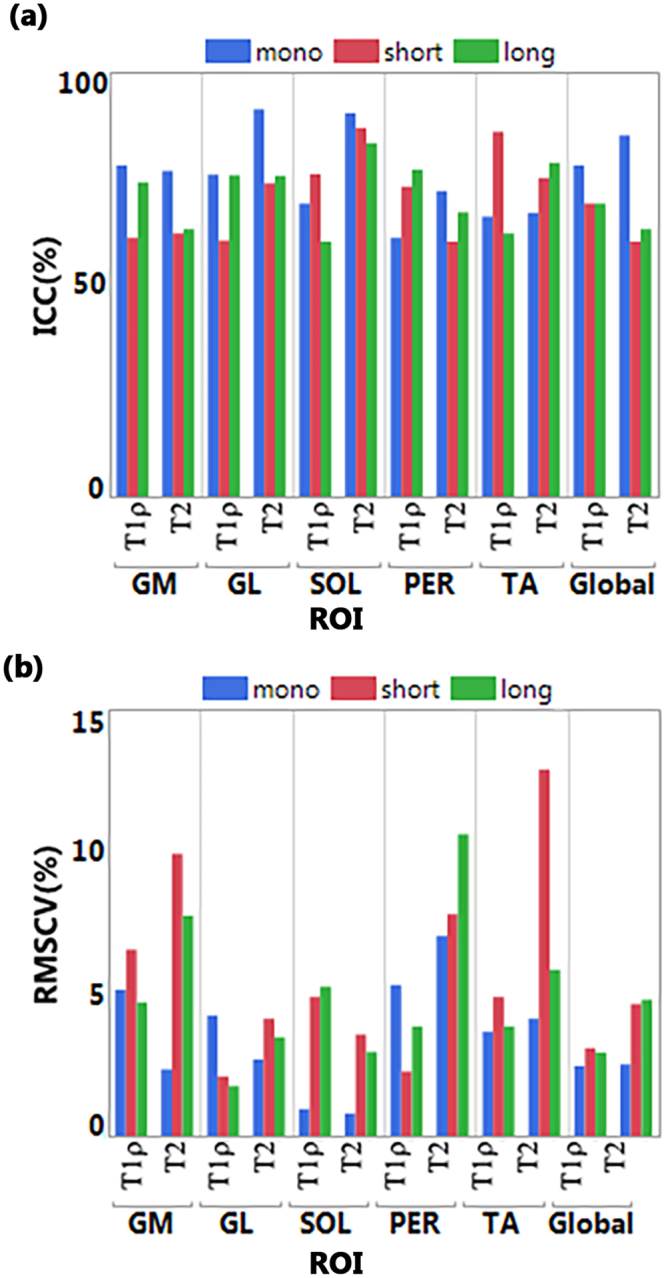
Repeatability study (**a**) ICC (**b**) RMSCV in different muscle ROIs.

Figure 6 shows the ICC and RMSCV across three participants. The ICC > 60% and RMSCV < 15% on all the regions show the good reliability and repeatability of this study.

## Discussion

In this paper, we presented a 3T MRI technique for *in-vivo*, bi-component T1ρ and T2 analysis of calf muscle. Five ROIs were defined in the muscle and T1ρ, and T2 relaxation times were measured in each ROI. The calf muscle chemical composition consists of intra- (~25%) and extracellular water (~75%), contractile proteins (~20%: myosin, actin, tropomyosin/troponin, myoglobin) and other components (~5%: salts, phosphates, ions, glycogen, and macronutrients). The short components are thought to be related to the tightly bound macromolecular (collagen, contractile proteins, and other components, etc.) and intracellular water compartments; while the long relaxation component corresponds mainly to the loosely bound water (extracellular/vascular). The results showed that biexponential fitting might better present and distinguish the different relaxation times in the muscle due to different water compartments. The estimated monoexponential relaxations were comparable to the other studies^3,13^. The monoexponential estimated T1ρ was higher than T2. To the best of our knowledge this comparison has not been reported for the calf muscle, however, our results trend are in agreement with other tissues such as articular cartilages^17–20^ in which T1ρ > T2.

The existence of three relaxation components was shown in previous studies using Carr-Purcell-Meiboom-Gill (CPMG) sequence^21,22^. Saab *et al*.^21^ reported the *in vivo* multi-component T2 relaxations in flexor digitorum profundus muscle while Cole *et al*.^22^ measured the T2 relaxation in rat muscle. These three components have been related to the hydration shell of macromolecules, intracellular water, and extracellular water, respectively^21–23^. In a study performed by Araujo *et al*.^24^ the existence of the biexponential relaxation behavior (e.g., an intermediate and a long components) has been confirmed for T2 relaxation time using a localized 2D-ISIS-CPMG sequence. However, no short component has been detected due to long TE’s used in the sequence^24^. In contrast, our method can measure a short component and provide 3D volumetric maps. Recently, Araujo *et al*.^2^ proposed a UTE sequence to measure the short T2 components. Only the short component was measured due to the short TEs used in this study. The total acquisition time to acquire one scan with 7 TE values was 7 min, 49 s. They measured T2 in only one thick (6 mm) slice while we acquired 3D volumetric scans. However, our method cannot detect very short T2 due to using TE of 3.78 ms in the readout.

Due to the SAR limitation in the T1ρ experiment, the longest TE/TSL in our study was 55 ms. Hence; our long component is close to the third component, and our short component is close to the first component calculated in Saab study. Moreover, the smaller slice thickness (2 ms) in our study in comparison with Saab study (10 mm) leads to lower partial volume effect (PVE).

To the best of our knowledge, biexponential T1_ρ_ measurement of muscle has only been done on animals. For example, a biexponential analysis of T1_ρ_ in rat muscles was reported by Yuan *et al*.^15^. The mono, short and long T1_ρ_ were measured as (~30–33 ms), (~9–11 ms) and (~37–41 ms), respectively. The short and long fractions were (~12–20%) and (~80–88%). 25 temporal points from TSL = 1 ms to 60 ms were acquired in this study at the cost of increasing the total acquisition time to ~30 minutes^15^. Our T_1ρ_ estimations using 10 TSLs acquired in 15 minutes scans are in good agreement with this study. The difference is probably due to the higher temporal resolution in Yuan study.

The selection of TR can affect the T1ρ and T2 estimation due to the T1 relaxation. We evaluated this effect using Bloch simulation. Our simulation showed that there is ~5% difference between the T1ρ or T2 estimation error with TR = 1500 ms and TR = 5000 ms. Considering the longer acquisition time of TR = 5000 ms, we considered this error as negligible.

The fatty infiltration of muscular occurring in some cases such as atrophy and muscular dystrophy does not affect the relaxation time since the fat signal was suppressed in the scans by exciting only the water with a binomial RF excitation pulse^25,26^.

Our study has some limitations. The biexponential condition of 4T_short_ < T_long_ can produce some bias. We chose this condition based on the suggestion in Juras study^27^.

Field inhomogeneities also affected the estimation since the spin-locking in T1_ρ_ imaging is very sensitive to B_0_ and B_1_ inhomogeneities. To compensate this effect, as described in our previous studies^26^, we used spin-lock phase alteration and a refocusing pulse for B_1_ and B_0_ compensation, respectively. In addition, the manual shimming was performed to further correct the field inhomogeneities. Our results showed a homogenous B_0_ (ΔB0 < ±5 Hz) and B_1_ changes less than ±50 Hz across ROIs. However, the compensation techniques used in this study may not be successful for scanning large volumes such as gluteus muscle or covering inhomogeneous regions such as arms and dorsal muscles. Moreover, the binomial RF excitation pulse is not robust in the presence of large inhomogeneities, and hence, the fatty infiltration may affect the estimation. Further studies in the large volumes muscles and, in the presence of fatty infiltration are warranted to evaluate the performance of our method.

The magic angle effect related to dipolar interactions of fiber orientation with respect to B_0_ may affect the T_1ρ_ and T_2_ values. The spins decay monoexponentially when the tissue’s orientation to B_0_ is about 55°^28^, though T_1ρ_ is relatively less sensitive than T_2_ to this effect^29^.

Finally, we expect an elevation in relaxation components due to a muscular disease such as muscle fibrosis. However, only a small number of asymptomatic participants were scanned in this study and further validation in patients with fibrosis is warranted.

In conclusion, in this study, we showed the feasibility of *in vivo* measurement of bi-exponential T1ρ and T2 relaxation of human calf muscle in clinically feasible scan times. Our method could potentially be used in intervention exercise studies or in studies of inflammatory myopathies or muscle fibrosis, permitting greater sensitivity and specificity via measurement of different water compartments and their fractions.

## Methods

### Monte Carlo Simulation

The signal decay during the spin-lock duration (TSL) or echo time (TE) can be defined as the summation of *M* exponential terms with different fractions (*a*_*i*_) and relaxation time constants (*T*_*i*_):

1 $${S}({t})={{S}}_{0}\sum _{{i}=1}^{{M}}{{a}}_{{i}}{{e}}^{-\frac{{t}}{{{T}}_{{i}}}}+{N}$$

where *S(t)* is the MRI signal intensity at time *t (*TSL in T1ρ and TE in T2 imaging), *S*_0_ is initial value, a_*i*_ is the fraction of *i* ^*th*^ exponential term with the assumption of $\sum _{i=1}^{M}{a}_{i}=1$, and *N* is the additive noise. Assuming *S*_0_ = 1, to express biexponential decay (*M* = 2), the equation can be written as:

2 $$S(t)={a}_{l}{e}^{-\frac{t}{{T}_{l}}}+{a}_{s}{e}^{-\frac{t}{{T}_{s}}}+N$$

where *T*_s_ and *T*_l_ are long and short relaxing components, respectively. The long (*a*_l_) and short (*a*_s_) fractions can be expressed in percentage as *F*_l_ = 100 × *a*_l_ *⁄(a*_l__+_ *a*_s_) and *F*_s_ = 100 × *a*_s_ *⁄ (a*_l__+_ *a*_s_), respectively. To estimate the relaxation time constants and their fractions, the MR signal must be acquired at several time points. Under given signal to noise ratio (SNR), the smaller number of points is desired to minimize the scan time. To determine the adequate range and number of points for successful estimation, Mont Carlo simulation^30^ was performed for 1000 random noise trail with normal distribution N(0,σ). The SNR was defined as SNR = 1/σ. The estimation errors were calculated for each noise trail as:

3 $$E=|\frac{{y}_{e}-{y}_{a}}{{y}_{a}}|$$

where *y*_*a*_ and *y*_*e*_ are the actual and estimated values, respectively. The average of errors in 1000 trial was reported in percentage as the Monte Carlo simulation result.

### *In-vivo* MRI acquisition

**Figure 7:**
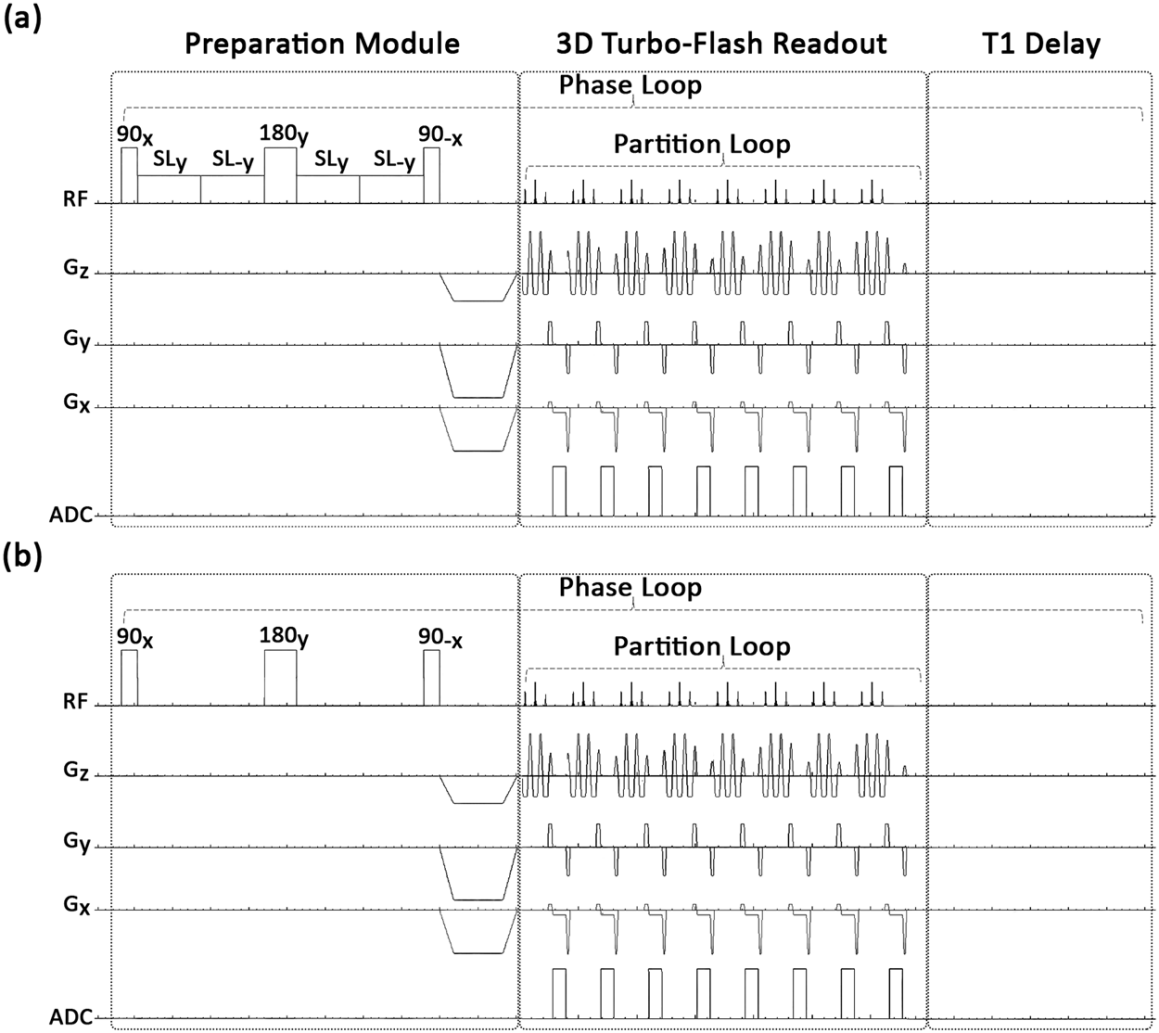
The imaging sequence timing diagram with (**a**) T_1ρ_ and (**b**) T_2_ preparation, 3D turbo-Flash readout, and T1 recovery delay. To compensate the effect of B_1_ inhomogeneities, the spin-lock pulse was divided into four segments with alternative phase. The refocusing pulse was applied between two pairs to compensate the B_0_ inhomogeneities. One phase line from all slices was acquired after applying the preparation module (partition loop). After a delay for T1 restoration, another preparation module was applied to acquire the next phase line (phase loop).

The study was approved by the institutional review board (IRB). All methods were performed in accordance with the relevant guidelines and regulations, and all of the participants signed a written informed consent prior to MRI scanning. Four females (age: 26 ± 3 years, BMI: 22 ± 1 kg/m^2^) and four male participants (age: 30 ± 3 years and BMI: 24 ± 2) with no signs of muscle pains or history of lower leg muscle injuries were recruited for this study. Additionally, follow-up scans were acquired from three participants two weeks after their first scan. 3D T1ρ and T2-weighted MR scans were taken on a 3T whole-body clinical MRI scanner (Prisma, Siemens Healthcare, Erlangen, Germany) with a 15-channel Tx/Rx knee coil (QED, Cleveland OH). 3D-Cartesian turbo-flash (TFL) sequence was used after T1ρ or T2 preparation module as readout followed by a delay for T1 restoration. The sequence timing diagram is shown in Fig. 7. Fat-suppressed T1ρ- and T2-weighted scans were acquired in the sagittal plane at 10 different TSL/TEs including 2, 4, 6, 8, 10, 15, 25, 35, 45, and 55 ms (Fig. 2). The total scans time to acquire both T1ρ and the T2-weighted data set was 29 min, 30 s. The sequence acquisition parameters were as follows: TR/TE = 1500 ms/3.78 ms, flip angle = 8°, field of view (FOV) = 140 mm^2^, matrix size 256 × 128 × 64, slice thickness = 2 ms, GRAPPA^31^ acceleration factor (AF) = 3. The spin-lock frequency (FSL) of 500 Hz was used in T1ρ preparation module.

### Biexponential T1ρ and T2 relaxation mapping

The T1ρ and T2 data were analyzed using an in-house program developed in MATLAB (R2017a, The MathWorks Inc., Natick, MA, USA). The mono- and biexponential models were applied pixel by pixel over five consecutive slices for each volunteer in the five ROIs (Fig. 2): Gastrocnemius Medialis (GM), Gastrocnemius Lateralis (GL), Soleus (SOL), Peroneus longus (PER), and Tibialis Anterior (TA).

In the final biexponential estimation the pixels where  4*T*_*short*_ < *T*_*long*_were excluded from the analysis^27^.

### Statistical analysis

The statistical analysis was performed using JMP statistical software (JMP®, Version 13 SAS Institute Inc., Cary, NC, 1989–2007). Wilcoxon rank sum test was applied to compare T1ρ and T2 relaxation components as well as gender difference. T1ρ and T2 relaxation components were also compared in different ROIs using the Kruskal-Wallis test.

The repeatability studies were performed on three participants by repeating the scans after two weeks. The coefficient of variation (CV) for each participant was calculated as

4 $$CV=\frac{{\sigma }}{{\mu }}$$

where µ and σ are the mean and standard deviation of the estimated components from two scans, respectively. The root-mean-squared CV (RMSCV) was then calculated across three subjects to evaluate the inter-subject repeatability:

5 $$RMSCV=\frac{\sqrt{\frac{{\sigma }_{1}^{2}+{\sigma }_{2}^{2}+{\sigma }_{3}^{2}}{3}}}{\frac{{\mu }_{1}+{\mu }_{2}+{\mu }_{3}}{3}}$$

Moreover, the intra-class correlation (ICC) was calculated as:

6 $$ICC=\frac{{\sigma }_{b}^{2}\,}{{\sigma }_{b}^{2}+{\sigma }_{w}^{2}}$$

where ${\sigma }_{b}^{2}$ and ${\sigma }_{w}^{2}$ are the between and within subjects variances, respectively.

### Data availability

The datasets generated during and/or analyzed during the current study are available from the corresponding author on reasonable request.

## References

[1] Carlier PG (2015). Skeletal muscle quantitative nuclear magnetic resonance imaging follow-up of adult Pompe patients. Journal of inherited metabolic disease.

[2] Araujo, E. C. A., Azzabou, N., Vignaud, A., Guillot, G. & Carlier, P. G. Quantitative ultrashort TE imaging of the short-T2 components in skeletal muscle using an extended echo-subtraction method. *Magnetic Resonance in Medicine*, **78**, 997-1008, doi:10.1002/mrm.26489 (2017). 10.1002/mrm.26489 27699843

[3] Rybak LD, Torriani M (2003). Magnetic Resonance Imaging of Sports-Related Muscle Injuries. Topics in Magnetic Resonance Imaging.

[4] Weber, M. A. *Magnetic Resonance Imaging of the Skeletal Musculature*. (Springer Berlin Heidelberg, 2013).

[5] Noseworthy, M. D. & Akbari, A. Mapping Skeletal Muscle Spin-Spin (T2) Relaxation[abstract]. *In: ISMRM 20th Annual Meeting & Exhibition* (2012).

[6] Franczak MB (2000). Spin‐Lock Magnetic Resonance Imaging of Muscle in Patients With Autosomal Recessive Limb Girdle Muscular Dystrophy. Journal of Neuroimaging.

[7] Maillard SM (2004). Quantitative assessment of MRI T2 relaxation time of thigh muscles in juvenile dermatomyositis. Rheumatology.

[8] Liu M (2006). Changes in muscle T2 relaxation properties following spinal cord injury and locomotor training. European journal of applied physiology.

[9] Polak JF, Jolesz FA, Adams DF (1988). Magnetic Resonance Imaging of Skeletal Muscle Prolongation of T1 and T2 Subsequent to Denervation. Investigative radiology.

[10] Iijima, Y. *et al*. Differences in fatty degeneration of rotator cuff muscles at different sites, as quantified by T2 mapping. *Journal of Orthopaedic Science* **22**, 281–284, https://doi.org/10.1016/j.jos.2016.11.016 (2017). 10.1016/j.jos.2016.11.016 27964874

[11] Wang L, Regatte RR (2015). T1ρ MRI of human musculoskeletal system. Journal of Magnetic Resonance Imaging.

[12] Wang L (2012). T1rho MRI of menisci and cartilage in patients with osteoarthritis at 3T. European journal of radiology.

[13] Hatakenaka M, Ueda M, Ishigami K, Otsuka M, Masuda K (2001). Effects of aging on muscle T2 relaxation time: difference between fast-and slow-twitch muscles. Investigative radiology.

[14] Hazlewood CF, Chang DC, Nichols BL, Woessner DE (1974). Nuclear Magnetic Resonance Transverse Relaxation Times of Water Protons in Skeletal Muscle. Biophysical Journal.

[15] Yuan J, Zhao F, Chan Q, Wang Y-XJ (2012). Observation of bi-exponential T1ρ relaxation of *in-vivo* rat muscles at 3T. Acta Radiologica.

[16] Qian Y, Williams AA, Chu CR, Boada FE (2010). Multicomponent T2* mapping of knee cartilage: technical feasibility *ex vivo*. Magnetic resonance in medicine.

[17] Regatte RR, Akella SV, Lonner J, Kneeland J, Reddy R (2006). T1ρ relaxation mapping in human osteoarthritis (OA) cartilage: comparison of T1ρ with T2. Journal of Magnetic Resonance Imaging.

[18] Pandit P, Talbott JF, Pedoia V, Dillon W, Majumdar S (2016). T1rho and T2 -based characterization of regional variations in intervertebral discs to detect early degenerative changes. Journal of orthopaedic research: official publication of the Orthopaedic Research Society.

[19] Zarins ZA (2010). Cartilage and meniscus assessment using T1rho and T2 measurements in healthy subjects and patients with osteoarthritis. Osteoarthritis and Cartilage.

[20] Stahl R (2009). T1rho, T2 and focal knee cartilage abnormalities in physically active and sedentary healthy subjects versus early OA patients—a 3.0-Tesla MRI study. European radiology.

[21] Saab G, Thompson RT, Marsh GD (1999). Multicomponent T2 relaxation of *in vivo* skeletal muscle. Magnetic resonance in medicine.

[22] Cole WC, Leblanc AD, Jhingran SG (1993). The origin of biexponential T2 relaxation in muscle water. Magnetic Resonance in Medicine.

[23] Gambarota G, Cairns BE, Berde CB, Mulkern RV (2001). Osmotic effects on the T2 relaxation decay of *in vivo* muscle. Magnetic Resonance in Medicine.

[24] Araujo, E C. A., Fromes, Y. & Carlier, Pierre G. New Insights on Human Skeletal Muscle Tissue Compartments Revealed by *In Vivo* T2 NMR Relaxometry. *Biophysical Journal* **106**, 2267–2274, https://doi.org/10.1016/j.bpj.2014.04.010 (2014). 10.1016/j.bpj.2014.04.010 PMC4052352 24853755

[25] Sharafi, A., Chang, G. & Regatte, R. R. Biexponential T2 relaxation estimation of human knee cartilage *in vivo* at 3T. *Journal of Magnetic Resonance Imaging*, 10.1002/jmri.25778 (2017). 10.1002/jmri.25778 PMC5711646 28561955

[26] Sharafi, A., Xia, D., Chang, G. & Regatte, R. R. Biexponential T1ρ relaxation mapping of human knee cartilage *in vivo* at 3 T. *NMR in Biomedicine*, 10.1002/nbm.3760 (2017). 10.1002/nbm.3760 PMC5597480 28632901

[27] Juras V (2014). Quantitative MRI analysis of menisci using biexponential T2* fitting with a variable echo time sequence. Magnetic resonance in medicine.

[28] Henkelman RM, Stanisz GJ, Kim JK, Bronskill MJ (1994). Anisotropy of NMR properties of tissues. Magnetic resonance in medicine.

[29] Li X (2011). Quantitative MRI using T 1ρ and T 2 in human osteoarthritic cartilage specimens: correlation with biochemical measurements and histology. Magnetic resonance imaging.

[30] Rubinstein, R. Y. & Kroese, D. P. *Simulation and the Monte Carlo method*. Vol. 707 (John Wiley & Sons, 2008).

[31] Griswold MA (2002). Generalized autocalibrating partially parallel acquisitions (GRAPPA). Magnetic resonance in medicine.

